# Supplementary figures and images for: Reassessing the genetic variability of Tectona grandis through high-throughput genotyping: Insights on its narrow genetic base
Source: PLoS One. 2023 Oct 26;18(10):e0285518. doi: 10.1371/journal.pone.0285518 (PMC10602281; doi:10.1371/journal.pone.0285518)

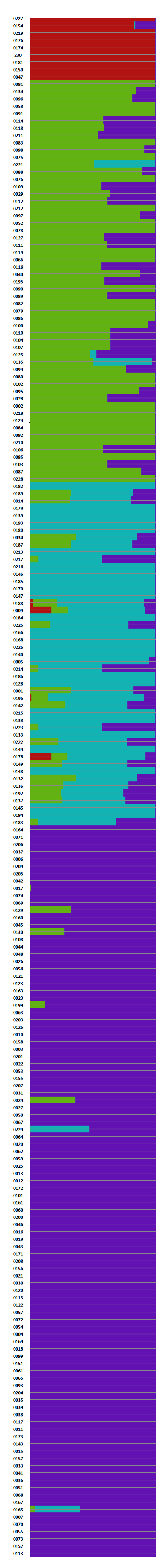

Supplement: S1 Fig — The numbers on the left of the image correspond to the identification of each genotype. (TIF) [file pone.0285518.s001.tif]
