# Supplementary material for: Reassessing the genetic variability of Tectona grandis through high-throughput genotyping: Insights on its narrow genetic base
Source: PLoS One. 2023 Oct 26;18(10):e0285518. doi: 10.1371/journal.pone.0285518 (PMC10602281; doi:10.1371/journal.pone.0285518)
Supplement: Attachment — Submitted filename: Response to Reviewers.pdf [file pone.0285518.s006.pdf]

**FROM: Thiago Alexandre Santana Gilio** , Corresponding Author

**TO:** Dr. Mehdi Rahimi

PlosOne Academic Editor

Dear Dr. Mehdi Rahimi

I am delighted to resubmit our original research article titled "Reassessing the Genetic Variability of Tectona grandis through High-Throughput Genotyping: Insights on its Narrow Genetic Base" for your consideration for publication in the Plos One journal, incorporating the necessary revisions as requested.

I emphasize again that our manuscript is the first to use sequencing genotyping in Tectona grandis, and through our study we concluded that the genetic diversity in this species is greater than that reported in other previously published manuscripts.

Outlined below are responses addressing the feedback provided by the Academic Editor and the Reviewer:

## Review responses to the Academic Editor

1. Please ensure that your manuscript meets PLOS ONE's style requirements, including those for file naming.

**Response -** Manuscript has been amended to meet journal style and file naming guidelines as requested.

2. **Response -** We have modified the acknowledgments section as requested to exclude the funding information.

Please amend the Financing Statement to the new version:

"This study was partially funded by the Coordination for the Improvement of Higher Education Personnel – Brazil (CAPES), by the Mato Grosso State Research Support Foundation (FAPEMAT) and by Teak Resources Company (TRC agroforestry) LTDA"

3. We note that the grant information you provided in the 'Funding Information' and 'Financial Disclosure' sections do not match. When you resubmit, please ensure that you provide the correct grant numbers for the awards you received for your study in the 'Funding Information' section.

**Response -** The financial disclosure will be revised before submission..

4-5. The data availability statement was changed and the DOI of the publication was inserted at the end of the manuscript (DOI:10.5281/zenodo.8171369)

6. We note that you have referenced (Keogh R. Teak (Tectona grandis) provenances of the Caribbean, Central America, Venezuela and Colombia. Unpublished Original document at FAO headquarters, Rome. 1978.) which has currently not yet been accepted for publication. Please remove this from your References and amend this to state in the body of your manuscript: (ie "Bewick et al. [Unpublished]") as detailed online in our guide for authors

**Response -** The highlighted reference was removed from the manuscript, without prejudice to the text.

7. Issues about the Figures.

**Response** - The highlighted figure was removed from the manuscript due to the copyright situation, without prejudice to the text.

8. Please include captions for your Supporting Information files at the end of your manuscript, and update any in-text citations to match accordingly. Please see our Supporting Information guidelines for more information: http://journals.plos.org/plosone/s/supporting-information.

**Response:** Supplemental file legends have been included in the supporting information at the end of the manuscript, as requested.

9. Please review your reference list to ensure that it is complete and correct. If you have cited papers that have been retracted, please include the rationale for doing so in the manuscript text, or remove these references and replace them with relevant current references. Any changes to the reference list should be mentioned in the rebuttal letter that accompanies your revised manuscript. If you need to cite a retracted article, indicate the article's retracted status in the References list and also include a citation and full reference for the retraction notice.

**Response**: the references have been refreshed, along with the update of all citation numbers throughout the manuscript.

The citation "(Keogh R. Teak (Tectona grandis) from the Caribbean, Central America, Venezuela and Colombia. Original document unpublished at FAO headquarters, Rome. 1978.)" was removed from the text without bias, taking into account the incorporation of other jointly used sources.

The subsequent references have been included in the Discussion section and the reference list to substantiate a point requested by the reviewer:

63. Reis CAF, Paludzyszyn Filho E. Estado da arte de plantios com espécies florestais de interesse para o Mato Grosso. Documentos - Embrapa Florestas. 2011 [cited 16 Aug 2023]. Available: https://www.infoteca.cnptia.embrapa.br/bitstream/doc/898075/1/Doc215.pdf

64. RAMALHO MAP, Santos JB dos, Pinto C, Souza EA de, Gonçalves FMA, Souza JC de. Genética na agropecuária. rev. Lavras: Ufla. 2004.

65. Carvalho J, SILVA MM de A, Medeiros MJL. Perda e conservação dos recursos genéticos vegetais. Documentos - Embrapa Algodão. 2009 [cited 16 Aug 2023].

Available:
https://www.infoteca.cnptia.embrapa.br/bitstream/doc/656849/1/DOC221.PDF

## Responses to the Reviewer

1. While the overall language and clarity are reasonable, there are some instances where the wording could be improved for better readability. The author must check the manuscript's grammatical and typological mistakes throughout the manuscripts.

Line 25: “the sequencings ware aligned” or “the sequencings were aligned”.
Line 480-481: clear the sentence “Based in our analyses true high thought genotyping by sequence with a representative pool of teak genotypes…”

**Response**: Thank you very much for your input. The highlighted grammatical errors have now been rectified.

2. Check the figure’s serial numbers.

Line 202 and 212: The figure number is same for both the pictures, i.e. Fig 3.

**Response:** The numbering of figures has been adjusted to align with the recent modifications.

3. The discussion thoroughly analyses and compares the findings with previous studies. However, it would be beneficial to include a more extensive discussion on the implications of the narrow genetic base of Tectona grandis. What are the potential consequences for the species' adaptability, resilience, and future breeding programs?

**Response:** Two paragraphs have been inserted in the Discussion on this point section.

4. The conclusion could be strengthened by succinctly summarising the essential findings and their implications. It would be helpful to restate the main objectives and emphasise the novelty and significance of the research.

**Response:** Conclusion section has been amended as requested to summarize the findings.

5. Follow the proper journal format for writing the citations in the text and the reference section of the manuscript. The author can use the PLOS template to prepare references. Some mistakes are
Line 109: “test (Keiding et al., 1986; Kjaer & Suangtho, 1995)), while those of some unregistered”
Line 133: “(https://zenodo.org/record/3962665#.Y7MxfezMKrM)”

**Response:** The manuscript citations have been changed to journal format.

The authors would like to seize this opportunity to confirm that all figures have been submitted for PACE digital diagnosis, as recommended.

We are confident that our manuscript is well-suited for publication in Plos One and will contribute significantly as a point of reference within the field. Additionally, it has not been previously published, nor is it presently under review for publication elsewhere. There are no conflicts of interest to declare.

We extend our gratitude for your thoughtful consideration of our submission.

Prof. Dr. Thiago Alexandre Santana Gilio and Dra. Isabela Vera dos Anjos
